# Supplementary material for: Revealing the high variability on nonconserved core and mobile elements of Austropuccinia psidii and other rust mitochondrial genomes
Source: PLoS One. 2021 Mar 11;16(3):e0248054. doi: 10.1371/journal.pone.0248054 (PMC7951889; doi:10.1371/journal.pone.0248054)
Supplement: S1 Fig — The accession numbers are in parentheses. The sequences were aligned using the MUSCLE method. For the phylogenetic tree construction, the statistical methods Maximum Likelihood, the Bootstrap method test with 1000 repetitions, and the Hasegawa—Kishino—Yano model were performed. The numbers above tree nodes represent the bootstrap support values. M. perniciosa was used as an out-group. (DOCX) [file pone.0248054.s001.docx]

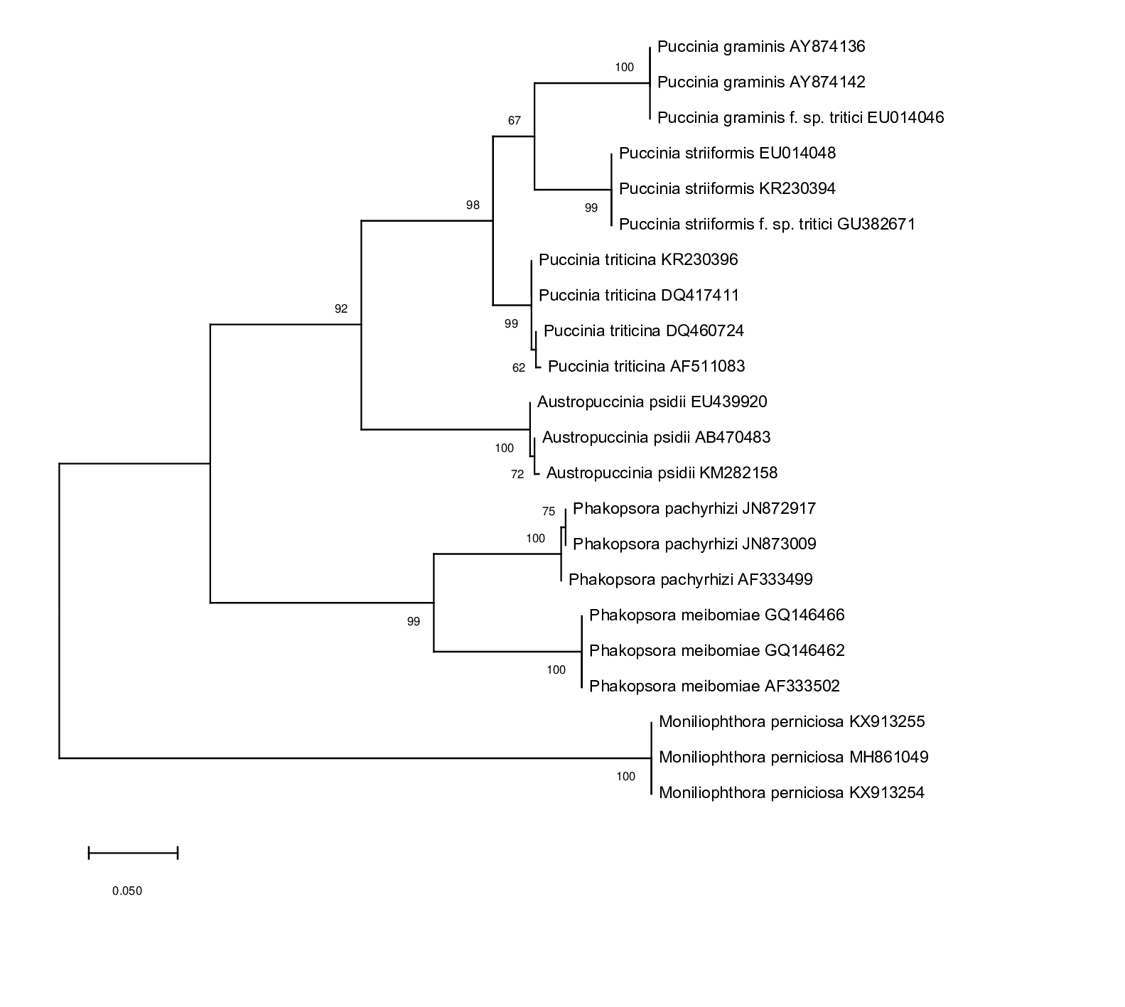
**S1 Figure.** Maximum likelihood phylogenetic tree of rust pathogens based on 18S rDNA partial sequences obtained from the GenBank nucleotide sequence database. The accession numbers are in parentheses. The sequences were aligned using the ClustalW method. For the phylogenetic tree construction, the statistical methods Maximum Likelihood, the Bootstrap method test with 1000 repetitions, and the Hasegawa - Kishino - Yano model were performed. The numbers above tree nodes represent the bootstrap support values. *M. perniciosa* was used as an out-group.
