# Supplementary material for: Revealing the high variability on nonconserved core and mobile elements of Austropuccinia psidii and other rust mitochondrial genomes
Source: PLoS One. 2021 Mar 11;16(3):e0248054. doi: 10.1371/journal.pone.0248054 (PMC7951889; doi:10.1371/journal.pone.0248054)
Supplement: S1 Table — (DOCX) [file pone.0248054.s002.docx]

**S1 Table. Conserved gene features of the *Austropuccinia psidii* MF-1 mitochondrial genome.**

| **Mitochondrial conserved genes** | **Location (nt)** | **Coding sequence length (bp)** | **Codon**  **Start  -Stop** | | **Strand** | **Interesting features** |
| --- | --- | --- | --- | --- | --- | --- |
| **Respiratory chain proteins** | |  |  |  |  |  |
| Complex I |  |  |  |  |  |  |
| *nad1* | 66972 – 67965 | 693 | AUG | UAG | + | contiguous and in phase with *orf162* |
| *nad2* | 1898 – 3098 | 1211 | AUG | UAG | + |  |
| *nad3* | 3111 – 3459 | 348 | AUG | UAA | + |  |
| *nad4* | 23546 – 24989 | 1441 | AUG | UAG | + |  |
| *nad4L* | 72106 – 72373 | 267 | AUG | UAG | + |  |
| *nad5* | 73659 – 75231 | 1560 | AUG | UUU | + |  |
| *nad6* | 65039 – 65642 | 603 | AUG | UAA | + |  |
| Complex III |  |  |  |  |  |  |
| *Cob* | join 31554 - 31755; 33377 - 33605 ; 34915 - 34975; 36403 - 36721; 38893 – 39404 | 1317 | AUG | UAA | + | I2 - group=ID  I3 and I4 -group=IA(5') |
| Complex IV |  |  |  |  |  |  |
| *cox1* | join 44699 - 44928; 47481 - 47594; 50065 - 50138; 51340 - 51568; 52783 - 52792; 53993 – 54076; 55666 - 55678; 56640 - 56727; 57759 - 57819; 58894 - 58927, 59991 - 60198; 61623 - 63057 | 2568 | AUG | UAA | + | group II – I1 and I2  group=IA – I5 and I12  group=IB – I3, I4, I7, I8, I9, I10 and I11  group=ID – I6 |
| *cox2* | join 12664 - 12976; 14240 - 14678 | 750 | AUG | UAA | + |  |
| *cox3* | 17117 - 17948 | 831 | AUG | UAA | + | alternative ATG start pos 17117 |
| Complex V |  |  |  |  |  |  |
| *atp6* | 9429 - 10197 | 768 | AUG | UAA | + |  |
| *atp8* | join 5984 – 6028;  8598 - 8696 | 98 | AUG | UAA | + |  |
| *atp9* | 85396-85618 | 222 | AUG | UAA | + | alternative ATG start pos 85321 |
| **Ribossomal proteins** | |  |  |  |  |  |
| *Rns* | 41966-43116 | - | - | - | + |  |
| *Rnl* | 82468-82863 | - | - | - | + |  |
| **Other protein** | |  |  |  |  |  |
| *rpn*B | 21603- 22584 | 981 | AUG | UAC | + |  |
