# Supplementary material for: Revealing the high variability on nonconserved core and mobile elements of Austropuccinia psidii and other rust mitochondrial genomes
Source: PLoS One. 2021 Mar 11;16(3):e0248054. doi: 10.1371/journal.pone.0248054 (PMC7951889; doi:10.1371/journal.pone.0248054)
Supplement: S2 Table — (DOCX) [file pone.0248054.s003.docx]

**S2 Table. Nonconserved ORFs (ncORFs) features of the *Austropuccinia psidii* MF-1 mitochondrial genome.**

| **ncORF** | **Start position** | **Stop position** | **Length (pb)** | **Strand** | **Start codon** | **Stop Codon** | **Target sequence** | **Characteristics** |
| --- | --- | --- | --- | --- | --- | --- | --- | --- |
| *orf99* | 86071 | 86371 | 300 | - | AUG | UAA | hypothetical protein | Intergenic ncORF |
| *orf100* | 65873 | 65573 | 300 | - | AUG | UAG | hypothetical protein | Intergenic ncORF;  in opposite strand of nad6 |
| *orf101* | 13482 | 13788 | 306 | + | AUG | UAG | LAGLIDADG endonuclease | Intronic ncORF (*cox2*-I1) |
| *orf104* | 85921 | 85606 | 315 | - | AUG | UAG | hypothetical protein | Intergenic ncORF;  in same sequence oposite strand of ATP9 |
| *orf115* | 76269 | 75921 | 348 | - | AUG | UAG | hypothetical protein | Intergenic ncORF |
| *orf116* | 27429 | 27078 | 351 | - | AUG | UAG | hypothetical protein | Intergenic ncORF;  TTG upstream: 27503 |
| *orf118_1* | 78695 | 78338 | 357 | - | AUG | UAG | hypothetical protein | Intergenic ncORF |
| *orf118_2* | 57819 | 58176 | 357 | + | UAU | UAA | LAGLIDADG endonuclease | Intronic ncORF (*cox1*-I9)  First aa- Tyr |
| *orf120* | 93262 | 92899 | 363 | - | AUG | UAA | hypothetical protein | Intergenic ncORF |
| *orf123_1* | 1084 | 712 | 372 |  | AUG | UAG | hypothetical protein | Intergenic ncORF |
| *orf123_2* | 68403 | 68031 | 372 | - | AUG | UAG | hypothetical protein | Intergenic ncORF |
| *orf125* | 19450 | 19072 | 378 | - | AUG | UAA | hypothetical protein | Intergenic ncORF |
| *orf128* | 69145 | 68758 | 387 | - | AUG | UAG | hypothetical protein | Intergenic ncORF |
| *orf130* | 28591 | 28984 | 393 | + | AUG | UAA | hypothetical protein | Intergenic ncORF |
| *orf132* | 79554 | 79155 | 399 | - | AUG | UAG | hypothetical protein | Intergenic ncORF |
| *orf153* | 64104 | 63642 | 462 | - | AUG | UAA | hypothetical protein | Intergenic ncORF;  TTG upstream: 64208 |
| *orf162* | 1412 | 1898 | 486 | + | AUG | UAG | hypothetical protein | Inside *nad2* gene |
| *orf166* | 40328 | 39827 | 501 | - | AUG | UAG | DEAD/DEAH box helicase dominio | Intergenic ncORF |
| *orf171* | 54079 | 54594 | 516 | + | GAA | UAA | LAGLIDADG endonuclease | Intronic ncORF (*cox1*-I9);  First aa- Glu; |
| *orf174* | 16270 | 15745 | 525 | - | AUG | UAA | hypothetical protein | Intergenic ncORF |
| *orf175* | 51881 | 52409 | 528 | + | AUG | UAA | LAGLIDADG endonuclease | Intronic ncORF (*cox1*-I4) |
| *orf192* | 81747 | 81168 | 579 | - | AUG | UAG | hypothetical protein | Intergenic ncORF |
| *orf205* | 26695 | 26077 | 618 | - | AUG | UAA | hypothetical protein | Intergenic ncORF ;  TTG upstream: 26703 |
| *orf208* | 30906 | 30279 | 627 | - | AUG | UAG | hypothetical protein | Intergenic ncORF |
| *orf241* | 33605 | 34331 | 726 | + | AGU | UAG | LAGLIDADG endonuclease | Intronic ncORF (*cob*-I2)  First aa – Ser |
| *orf252* | 40283 | 41042 | 758 | + | AUG | UAG | ribosomal protein S3 | Intergenic ncORF |
| *orf311* | 55677 | 56613 | 936 | + | UGA | UAG | LAGLIDADG endonuclease | Intronic ncORF (*cox1*-I7);  Codon alternative para UGA para Trp |
| *orf319* | 58927 | 59887 | 960 | + | AUA | UAA | LAGLIDADG endonuclease | Intronic ncORF (*cox1*-I10)  First aa – Ile |
| *orf337* | 56727 | 57741 | 1014 | + | CAA | UAA | LAGLIDADG endonuclease | Intronic ncORF (*cox1*-I8)  First aa – Gln |
| *orf363* | 50139 | 51231 | 1092 | + | GGU | UAA | LAGLIDADG endonuclease | Intronic ncORF (*cox1*-I3)  First aa- Gly |
| *orf669* | 6091 | 8101 | 2010 | + | AUG | UAA | Reverse Transcriptase | Intronic ncORF  (*atp8* I1) |
| *orf688* | 47597 | 49664 | 2067 | + | GCC | UAA | Reverse Transcriptase | Intronic ncORF (*cox1*-I2)  First aa- Gly |
| *orf717* | 44930 | 47083 | 912 | + | ACG | UAA | Reverse Transcriptase | Intronic ncORF(*cox1*-I1)  First aa- Thr |
