# Supplementary material for: Revealing the high variability on nonconserved core and mobile elements of Austropuccinia psidii and other rust mitochondrial genomes
Source: PLoS One. 2021 Mar 11;16(3):e0248054. doi: 10.1371/journal.pone.0248054 (PMC7951889; doi:10.1371/journal.pone.0248054)
Supplement: S3 Table — (DOCX) [file pone.0248054.s004.docx]

**S3 Table.** Nonconserved ORFs (ncORFs) features in mtDNA rust pathogens.

| **ncORF** | **Start position** | **Stop position** | **Length (pb)** | **Strand** | | **Start codon** | **Stop Codon** | **Target sequence** | **Characteristics** |
| --- | --- | --- | --- | --- | --- | --- | --- | --- | --- |
| ***Phakopsora meibomiae* Puerto_Rico** | | | | | | | | | |
| *orf105* | 1950 | 1632 | 318 | - | | AUG | UAG | hypothetical protein | Intergenic ncORF |
| *orf111_1* | 7632 | 7296 | 336 | - | | AUG | UAG | hypothetical protein | Intergenic ncORF |
| *orf111_2* | 14642 | 14308 | 336 | - | | AUG | UAG | hypothetical protein | Intergenic ncORF |
| *orf119* | 362 | 2 | 360 | - | | AUG | UCA | hypothetical protein | Intergenic ncORF |
| *orf191* | 18269 | 18843 | 576 | + | | AUG | UAG | ribosomal protein S3 | Intergenic ncORF |
| *orf241* | 15960 | 16685 | 725 | + | | AGU | UAG | LAGLIDADG endonuclease | Intronic ncORF (*cob* - I1)  First aa – Ser |
| *orf255* | 23784 | 24552 | 768 | + | | ACC | UAA | LAGLIDADG endonuclease | Intronic ncORF (*cox1*-I4)  First aa=Thr |
| ***Phakopsora pachyrhizi* Taiwan_72-1** | | | | | | | | | |
| *orf101* | 31349 | 31655 | 306 | + | | AUG | UAA | hypothetical protein | Intergenic ncORF |
| *orf106* | 351 | 30 | 321 | - | | AUG | UAA | hypothetical protein | Intergenic ncORF |
| *orf186* | 17626 | 18186 | 560 | + | | AUG | UAA | ribosomal protein S3 | Intergenic ncORF |
| *orf241* | 15515 | 16240 | 725 | + | | AGU | UAG | hypothetical protein | Intronic ncORF (*cob* - I1)  First aa – Ser |
| *orf260* | 23052 | 23836 | 784 | + | | UCC | UAA | LAGLIDADG endonuclease | Intronic ncORF (*cox1* -  I4)  First aa – Thr |
| *orf370* | 20288 | 21400 | 1112 | + | | GGU | UAA | LAGLIDADG endonuclease | Intronic ncORF (*cox1* - I1)  First aa – Gly |
| ***Puccinia graminis f.* sp. *tritici*** | | | | | | | | | |
| *orf100* | 57717 | 57415 | 303 | - | | AUG | UAG | hypothetical protein | Intergenic ncORF  overlapping region with *cox2* and *cox2-*E*2*, opposite reading direction |
| *orf101* | 64360 | 64055 | 306 | - | | AUG | UAG | hypothetical protein | Intergenic ncORF contiguous with *nad4* and *nad4-*E1 in opposite reading direction |
| *orf102* | 71144 | 70836 | 309 | - | | AUG | UAA | hypothetical protein | Intergenic ncORF |
| *orf114* | 45171 | 44827 | 345 | - | | AUG | UAA | hypothetical protein | Intergenic ncORF |
| *orf120* | 52682 | 52320 | 363 | - | | AUG | UAG | hypothetical protein | Intergenic ncORF |
| *orf124* | 76977 | 76603 | 375 | - | | AUG | UAA | hypothetical protein | Intergenic ncORF |
| *orf154* | 15987 | 15523 | 465 | - | | AUG | UAG | hypothetical protein | Intergenic ncORF Overlapping region with *nad1,* opposite reading direction |
| *orf174* | 6211 | 6735 | 525 | + | | AUG | UAG | LAGLIDADG endonuclease | Intronic ncORF (*cox1*-I2) |
| *orf202* | 28805 | 28197 | 609 | - | | AUG | UAA | hypothetical protein | Intergenic ncORF |
| *orf210* | 71585 | 72217 | 633 | + | | AGU | UAA | LAGLIDADG endonuclease | Intronic ncORF (*cob*-I1)  First aa – Ser |
| *orf251_1* | 41948 | 41193 | 756 | - | | AUG | UAA | hypothetical protein | Intergenic ncORF |
| *orf251_2* | 78421 | 79176 | 756 | + | | AUG | UAG | hypothetical protein | Intergenic ncORF |
| *orf269* | 9417 | 10226 | 810 | + | | ACC | UAA | LAGLIDADG endonuclease | Intronic ncORF (*cox1*-I5)  First aa – Thr |
| *orf321* | 8146 | 9111 | 966 | + | | AUA | UAA | LAGLIDADG endonuclease | Intronic ncORF (*cox1*-I4)  First aa – Ile |
| *orf323* | 64859 | 65830 | 972 | + | | GUG | UAA | hypothetical protein | Intronic ncORF (*nad4*-I1)  First aa – Val |
| *orf357* | 6952 | 8025 | 1074 | + | | CAA | UAA | LAGLIDADG endonuclease | Intronic ncORF (*cox1*-I3)  First aa – Gln |
| *orf367* | 1465 | 2568 | 1104 | + | | GGU | UAA | LAGLIDADG endonuclease | Intronic ncORF (*cox1*-I1)  First aa – Gly |
| ***Puccinia striiformis* PST-78** | | | | | | | | | |
| *orf102* | 13286 | 12978 | 309 | - | | AUG | UAA | hypothetical protein | Intergenic ncORF |
| *orf117* | 1046 | 693 | 354 | - | | AUG | UAA | hypothetical protein | Intergenic ncORF |
| *orf134* | 61066 | 60662 | 405 | - | | AUG | UAA | hypothetical protein | Intergenic ncORF |
| *orf138* | 66319 | 66732 | 414 | + | | AUG | GAU | hypothetical protein | Contiguous and in phase with *nad2* |
| *orf155* | 53320 | 53787 | 468 | + | | AUG | UAA | hypothetical protein | Intergenic ncORF |
| *orf235* | 16724 | 17431 | 708 | + | | AUG | UAA | LAGLIDADG endonuclease | Intronic ncORF (*cob*-I1) |
| *orf263* | 21732 | 22523 | 792 | + | | AUG | UAA | ribosomal protein S3 | Intergenic ncORF |
| *orf278* | 34605 | 35441 | 837 | + | | ACU | UAA | LAGLIDADG endonuclease | Intronic ncORF (*cox1*-I6)  First aa – Thr |
| *orf312* | 31986 | 32924 | 939 | + | | AGA | UAG | LAGLIDADG endonuclease | Intronic ncORF (*cox1*-I4)  First aa –Arg |
| *orf318* | 33292 | 34248 | 957 | + | | AUG | UAG | LAGLIDADG endonuclease | Intronic ncORF (*cox1*-I5) |
| *orf384* | 26559 | 27713 | 1155 | + | | GGU | UAA | LAGLIDADG endonuclease | Intronic ncORF (*cox1*-I1)  First aa – Gly |
| *orf600* | 9935 | 11737 | 1803 | + | | AUG | UAA | hypothetical protein | Intronic ncORF (*nad4*-I1) |
| ***Puccinia triticina* 1-1BBBD-race-1** | | | | | | | | | |
| *orf105* | 6743 | 6426 | 318 | - | | AUG | UAG | hypothetical protein | Overlapping region with  *orf836*, opposite reading direction |
| *orf107* | 58643 | 58320 | 324 | | - | AUG | UAG | hypothetical protein | Intergenic ncORF |
| *orf108* | 40845 | 40519 | 327 | | - | AUG | UAA | hypothetical protein | Intergenic ncORF |
| *orf113* | 59427 | 59086 | 342 | | - | AUG | UAG | hypothetical protein | Intergenic ncORF |
| *orf120* | 13537 | 13175 | 363 | - | | AUG | UAA | hypothetical protein | Intergenic ncORF |
| *orf148* | 50674 | 50228 | 447 | | - | AUG | UAA | hypothetical protein | Intergenic ncORF |
| *orf149* | 36586 | 36137 | 450 | | - | AUG | UAG | hypothetical protein | Intergenic ncORF |
| *orf160* | 62246 | 61764 | 483 | - | | AUG | UAG | hypothetical protein | Intergenic ncORF |
| *orf162* | 38319 | 37831 | 489 | | - | AUG | UAA | hypothetical protein | Intergenic ncORF |
| *orf216* | 2522 | 1872 | 651 | | - | AUG | UAA | hypothetical protein | Overlapping region with *cox3*, opposite reading direction |
| *orf251* | 19495 | 20250 | 756 | | + | AUG | UAG | ribosomal protein S3 | Intergenic ncORF |
| *orf266* | 13978 | 14778 | 801 | | + | AGU | UAA | LAGLIDADG endonuclease | Intronic ncORF (*cob*-I1)  First aa – Ser |
| *orf269* | 31596 | 32405 | 810 | | + | ACC | UAA | LAGLIDADG endonuclease | Intronic ncORF (*cox1*-I7)  First aa - Thr |
| *orf311* | 27996 | 28931 | 936 | | + | UGA | UAG | LAGLIDADG endonuclease | Intronic ncORF (*cox1*-I4)  First aa – Trp |
| *orf321* | 30326 | 31291 | 966 | | + | AUA | UAA | LAGLIDADG endonuclease | Intronic ncORF (*cox1*-I6)  First aa – Ile |
| *orf357* | 29132 | 30205 | 1074 | | + | CAA | UAA | LAGLIDADG endonuclease | Intronic ncORF (*cox1*-I5)  First aa – Gln |
| *orf366* | 23681 | 24781 | 1101 | | + | GGU | UAA | LAGLIDADG endonuclease | Intronic ncORF (*cox1*-I1)  First aa – Gly |
| *orf836* | 6719 | 9226 | 2508 | | + | AUG | CCU | hypothetical protein | Contiguous and in phase with *nad4* |
