# Supplementary material for: Revealing the high variability on nonconserved core and mobile elements of Austropuccinia psidii and other rust mitochondrial genomes
Source: PLoS One. 2021 Mar 11;16(3):e0248054. doi: 10.1371/journal.pone.0248054 (PMC7951889; doi:10.1371/journal.pone.0248054)
Supplement: S4 Table — (DOCX) [file pone.0248054.s005.docx]

**Table S4.** Number of LAGLIDADG endonucleases and intron types present in introns of six genes gene in mtDNA of rust pathogens.

| **Rust Pathogen** | ***atp8*** | ***cob*** | ***cox1*** | ***cox2*** | ***nad4*** | ***nad5*** |
| --- | --- | --- | --- | --- | --- | --- |
| ***Austropuccinia psidii*** | | | | | | |
| LAGLIDADG endonuclease |  | 1 | 9 | 1 |  |  |
| group=IA |  | 3 | 2 | 1 |  |  |
| group=IB |  | 1 | 7 |  |  |  |
| group=ID |  | 1 | 1 |  |  |  |
| group=II |  |  | 2 |  |  |  |
| ***Phakopsora meibomiae*** | | | | | | |
| LAGLIDADG endonuclease |  | 1 | 1 |  |  |  |
| group=IA |  |  |  |  |  |  |
| group=IB |  |  | 2 |  |  |  |
| group=ID |  |  | 1 |  |  |  |
| group=II |  |  |  |  |  |  |
| ***Phakopsora pachyrhizi*** | | | | | | |
| LAGLIDADG endonuclease |  |  | 2 |  |  |  |
| group=IA |  |  |  |  |  |  |
| group=IB |  |  | 2 |  |  |  |
| group=ID |  | 1 | 1 |  |  |  |
| group=II |  |  |  |  |  |  |
| ***Puccinia graminis*** | | | | | | |
| LAGLIDADG endonuclease |  | 1 | 5 |  |  |  |
| group=IA |  |  |  | 1 |  |  |
| group=IB |  |  | 4 |  |  | 2 |
| group=ID |  |  |  |  |  |  |
| group=II |  |  |  |  | 1 |  |
| ***Puccinia striiformis*** | | | | | | |
| LAGLIDADG endonuclease |  | 1 | 3 |  |  |  |
| group=IA |  | 1 |  | 1 |  |  |
| group=IB |  |  | 4 |  |  | 1 |
| group=ID |  |  | 1 |  |  |  |
| group=II |  |  |  |  | 1 |  |
| ***Puccinia triticina*** | | | | | | |
| LAGLIDADG endonuclease |  | 1 | 5 |  |  |  |
| group=IA |  | 1 |  | 1 |  |  |
| group=IB |  |  | 5 |  |  | 1 |
| group=ID |  |  | 1 |  |  |  |
| group=II |  |  |  |  |  |  |
