# Supplementary material for: Revealing the high variability on nonconserved core and mobile elements of Austropuccinia psidii and other rust mitochondrial genomes
Source: PLoS One. 2021 Mar 11;16(3):e0248054. doi: 10.1371/journal.pone.0248054 (PMC7951889; doi:10.1371/journal.pone.0248054)
Supplement: S5 Table — (DOCX) [file pone.0248054.s006.docx]

**S5 Table.** Features of introns characterized in mtDNA of rust pathogens.

| **Rust pathogen** | **Intron** | **Intron type** | **Intron length (bp)** | **Intronic ORF type a** | **Intron ORF length (aa)** |
| --- | --- | --- | --- | --- | --- |
| ***A. psidii*** | *atp8 -* I1 |  |  | RT | 670 |
|  | *cob* - I1 | group=IB (5’) | 1622 | NI | - |
|  | *cob* -I2 | group=ID | 1310 | L | 254 |
|  | *cob* –I3 | group=IB (5’) | 1427 | NI | - |
|  | *cob* –I4 | group=IA (5’) | 2173 | NI | - |
|  | *cox1* –I1 | group=II | 2553 | RT | 304 |
|  | *cox1* –I2 | group=II | 2469 | RT | 689 |
|  | *cox1* –I3 | group=IB | 1202 | L | 364 |
|  | *cox1* –I4 | group=IB | 1214 | L | 176 |
|  | *cox1* –I5 | group=IA | 1200 | NI |  |
|  | *cox1* –I6 | group=ID | 1589 | L | 172 |
|  | *cox1* –I7 | group=IB | 963 | L | 312 |
|  | *cox1* –I8 | group=IB | 1032 | L | 320 |
|  | *cox1* –I9 | group=IB | 1075 | L | 119 |
|  | *cox1* –I10 | group=IB | 1064 | L | 320 |
|  | *cox1* –I11 | group=IB(3') | 1425 | NI | - |
|  | *cox1* –I12 | group=IA | 1236 | NI | - |
|  | *cox2* –I1 | group=IA (5’) | 1262 | NI | - |
| ***P. meibomiae*** | *cob*  I1 | * | 1492 | L | 341 |
|  | *cox1* –I1 | group=IB | 1129 | NI | - |
|  | *cox1* –I2 | group=ID | 512 | NI | - |
|  | *cox1* –I3 | group=IB | 347 | NI | - |
|  | *cox1* –I4 | group=IB | 1126 | L | 256 |
| ***P.  pachyrhizi*** | *cob*  I1 | group=ID | 1336 | L | 242 |
|  | *cox1* –I1 | group=IB | 1119 | L | 370 |
|  | *cox1* –I2 | group=ID | 562 | NI | - |
|  | *cox1* –I3 | group=IB | 363 | NI | - |
|  | *cox1* –I4 | group=IB(3') | 1333 | L | 360 |
| ***P. graminis*** | *cob*  I1 | * | 1481 | L | 311 |
|  | *cob –* I2 | group=IA | 2024 | NI | - |
|  | *cox1* –I1 | group=IB | 1207 | L | 368 |
|  | *cox1* –I2 | group=IB | 4097 | L | 175 |
|  | *cox1* –I3 | group=IB | 1101 | L | 358 |
|  | *cox1* –I4 | group=IB | 1064 | L | 322 |
|  | *cox1* –I5 | * | 1125 | L | 270 |
|  | *cox2* –I1 | group=IA | 1186 | NI | - |
|  | *nad4* – I1 | group=II | 2376 | HP | 324 |
|  | *nad5* I1 | group=IB | 1165 | NI | - |
|  | *nad5* I2 | group=IB | 1405 | NI | - |
| ***P. striiformis*** | *cob* I1 | * | 1535 | L | 264 |
|  | *cob -* I2 | group=IA | 2198 | NI | - |
|  | *cox1* –I1 | group=IB | 1260 | L | 385 |
|  | *cox1* –I2 | group=IB(5') | 2025 | NI | - |
|  | *cox1* –I3 | group=ID | 1809 | NI | - |
|  | *cox1* –I4 | group=IB(3') | 1058 | L | 313 |
|  | *cox1* –I5 | group=IB(3') | 1174 | L | 319 |
|  | *cox1*–I6 | * | 1163 | L | 279 |
|  | *cox2* –I1 | group=IA | 1112 | NI | - |
|  | *nad4* – I1 | group=II | 2422 | HP | 621 |
|  | *nad5* I1 | group=IB | 1680 | NI | - |
| ***P. triticina*** | *cob* I1 | * | 1484 | L | 270 |
|  | *cob –* I2 | group=IA | 1842 | NI | - |
|  | *cox1* –I1 | group=IB | 1207 | L | 367 |
|  | *cox1* –I2 | group=IB | 1231 | NI | - |
|  | *cox1* –I3 | group=ID | 1544 | NI | - |
|  | *cox1* –I4 | group=IB(3') | 1049 | L | 312 |
|  | *cox1* –I5 | group=IB | 1101 | L | 358 |
|  | *cox1*–I6 | group=IB | 1063 | L | 322 |
|  | *cox1*–I7 | * | 1127 | L | 270 |
|  | *cox2* –I1 | group=IA | 1165 | NI | - |
|  | *nad5*  I1 | group=IB | 1413 | NI | - |

* no intron type identified

a Intronic ORF types:  L - LAGLIDADG endonuclease; HP - hypothetical protein; RT – reverse transcriptase; NI – Non identified.
