# Supplementary material for: Revealing the high variability on nonconserved core and mobile elements of Austropuccinia psidii and other rust mitochondrial genomes
Source: PLoS One. 2021 Mar 11;16(3):e0248054. doi: 10.1371/journal.pone.0248054 (PMC7951889; doi:10.1371/journal.pone.0248054)
Supplement: S6 Table — (DOCX) [file pone.0248054.s007.docx]

**S6 Table.** Nonconserved ORFs (ncORFs) in mtDNA *Austropuccinia psidii* MF-1 shared by other rust pathogens.

| ***A. psidii*** **ncORF** | ***P. meibomiae*** | ***P. pachyrhizi*** | ***P. graminis*** | ***P. striiformis*** | ***P. triticina*** |
| --- | --- | --- | --- | --- | --- |
| *orf99* |  |  |  |  |  |
| *orf100* |  |  |  |  |  |
| *orf101* |  |  |  |  |  |
| *orf104* |  |  |  |  |  |
| *orf115* |  |  |  |  |  |
| *orf116* |  |  |  |  |  |
| *orf118_1* | 1e-94 (*orf119)* | 2e-93 *(orf106)* | 1e-162 (*orf202*) |  |  |
| *orf118_2* |  |  |  |  |  |
| *orf120* |  |  |  |  |  |
| *orf123_1* |  |  |  |  |  |
| *orf123_2* |  |  | 3e-53 (*orf154*) |  |  |
| *orf125* |  |  |  |  |  |
| *orf128* |  |  |  |  |  |
| *orf130* |  |  |  |  |  |
| *orf132* |  |  |  |  |  |
| *orf153* |  |  |  |  |  |
| *orf162* | 0.0 |  |  |  |  |
| *orf166* |  |  |  |  |  |
| *orf171* |  |  |  |  |  |
| *orf174* |  |  | 0.0 (*orf174*) |  |  |
| *orf175* |  |  |  |  |  |
| *orf192* |  |  |  |  |  |
| *orf205* |  |  |  |  |  |
| *orf208* |  |  |  |  |  |
| *orf241* | 0,0 (*orf241*) | 0.0 (*orf241*) | 0.0 (*orf210*) | 0.0 (*orf235*) | 0.0 *(orf266)* |
| *orf252* | 9e-44 (*orf191*) |  | 0.0 (*orf252*) | 0.0 (*orf263*) | 0.0 (*orf252*) |
| *orf311* |  |  |  | 0.0 (*orf312*) | 0.0 (*orf311*) |
| *orf319* |  |  | 1.0 (*orf321*) |  | 0.0(*orf321*) |
| *orf337* |  |  | 0.0 (*orf357*) | 2e-92 (*orf318)* | 0.0 (*orf357*) |
| *orf363* |  | 0.0 (*orf370*) | 0.0 (*orf367*) | 9e-162 *(orf384)* |  |
| *orf669* |  |  |  |  |  |
| *orf688* |  |  |  |  |  |
| *orf717* |  |  |  |  |  |

Values of *e-value* obtained by blastn using the NCBI database.

Corresponding ncORFs from rust pathogens.
