# Supplementary material for: Revealing the high variability on nonconserved core and mobile elements of Austropuccinia psidii and other rust mitochondrial genomes
Source: PLoS One. 2021 Mar 11;16(3):e0248054. doi: 10.1371/journal.pone.0248054 (PMC7951889; doi:10.1371/journal.pone.0248054)
Supplement: S7 Table — (DOCX) [file pone.0248054.s008.docx]

**S7 Table.** Conserved domain in unknown function proteins found in *Austropuccinia psidii.*

| **ncORF** | **Number of domains** | **Position (bp)** | **Domain size (pb)** | **Identification** | **Description** | **Superfamily** |
| --- | --- | --- | --- | --- | --- | --- |
| ***orf174*** | 1 | 41 - 87 | 46 | PRK05582 | Like DNA topoisomerase I | cl27598 |
| ***orf205*** | 2 | 18 – 68 | 50 | PF01328 | Like Peroxidase, family 2 | cl03166 |
|  |  | 147 - 204 | 57 | PF09843 | DUF2070 Predicted membrane protein | cl26595 |
| ***orf208*** | 1 | 3 – 136 | 133 | PF09843 | DUF2070 -Predicted membrane protein | cl26595 |
