# Supplementary material for: Revealing the high variability on nonconserved core and mobile elements of Austropuccinia psidii and other rust mitochondrial genomes
Source: PLoS One. 2021 Mar 11;16(3):e0248054. doi: 10.1371/journal.pone.0248054 (PMC7951889; doi:10.1371/journal.pone.0248054)
Supplement: Attachment — Submitted filename: Almeida et al - Response Letter.docx [file pone.0248054.s010.docx]

February 16, 2021

# **Letter Response**

Dear Academic Editor

Dr. Minou Nowrousian

PlosOne

This is a summary of the second revision made in the manuscript entitled “Revealing the high variability on nonconserved core and mobile elements of *Austropuccinia psidii* and other rust mitochondrial genomes” (PONE-D-20-26480R1). We are grateful for the contributions made by you. We accepted all suggestion find in the editor’ comment enclosed.

We look forward to hearing from you.

Best regards,

Maria Carolina Quecine

**Response to Editor**

We are grateful for the opportunity to resubmit again our manuscript. All mistakes were corrected in the manuscript.

1. Line 24: Please change "fungi rusts" to "rust fungi" (and make sure that this is the case throughout the text).

**R: We replaced “fungi rusts” to “rust fungi” in all manuscript.**

2. Line 104: should be "through" (not though)?

**R: Certainly, the correct spelling is “through”. It was corrected in the manuscript**

3. Line 272: Sentence is not quite clear. Should it be something like "in contrast to the conserved core genes, the ncORFs were found on both strands"?

**R: We agree that the sentence “The ncORFs diferentlly of conserved core genes were found in both strand” was confusing and it was replaced by “In contrast to the conserved core genes, the ncORFs were found on both strands” as suggested the editor.**

4. Lines 329-330: M. perniciosa should be in italics.

**R: We put in italics the name *M. perniciosa***

5. Line 356: Please correct spelling mistakes in "mtDNA", "size" and "occur".
6. Please check the text again for other spelling errors etc.

**R: We verified carefully the whole manuscript and all mistakes were corrected.**

We hope to have answered all questions,

Sincerely,

Maria Carolina Quecine
